# The hypoxic tumour microenvironment

**DOI:** 10.1038/s41389-017-0011-9

**Published:** 2018-01-24

**Authors:** Varvara Petrova, Margherita Annicchiarico-Petruzzelli, Gerry Melino, Ivano Amelio

**Affiliations:** 1 Medical Research Council, Toxicology Unit, Leicester University, Hodgkin Building, Lancaster Road, P.O. Box 138, Leicester, LE1 9HN UK; 2 Biochemistry Laboratory, IDI-IRCCS, Rome, Italy; 3 Department of Experimental Medicine and Surgery, University of Rome “Tor Vergata”, 00133 Rome, Italy

## Abstract

Cancer progression often benefits from the selective conditions present in the tumour microenvironment, such as the presence of cancer-associated fibroblasts (CAFs), deregulated ECM deposition, expanded vascularisation and repression of the immune response. Generation of a hypoxic environment and activation of its main effector, hypoxia-inducible factor-1 (HIF-1), are common features of advanced cancers. In addition to the impact on tumour cell biology, the influence that hypoxia exerts on the surrounding cells represents a critical step in the tumorigenic process. Hypoxia indeed enables a number of events in the tumour microenvironment that lead to the expansion of aggressive clones from heterogeneous tumour cells and promote a lethal phenotype. In this article, we review the most relevant findings describing the influence of hypoxia and the contribution of HIF activation on the major components of the tumour microenvironment, and we summarise their role in cancer development and progression.

## Hypoxia and hypoxia-inducible factors

The major components of the tumour microenvironment (TME) are blood vessels, lymphatic vessels, fibroblasts, immune cells and chemico-physical components such as the extracellular matrix (ECM)^1^. The functional and physical interaction of these elements with cancer cells determines clinical outcomes. During tumour development and progression, cancer and stromal cells often have restricted access to nutrients and oxygen. Most solid tumours indeed have regions permanently or transiently subjected to hypoxia because of aberrant vascularisation and a poor blood supply^2^. The hypoxic response is mainly ascribed to hypoxia-inducible factors (HIFs). HIF-dependent signalling can promote the adaptation and selection of both cancer and stromal cells to the surrounding conditions, thus promoting changes that favour cancer progression. The HIF family of transcription factors includes HIF1, HIF2 and HIF3. These factors all contain an oxygen-sensitive HIF-α subunit (HIF1-α, HIF2-α or HIF3-α, respectively), which dimerises with the constitutively expressed HIF1-β subunit^3^. HIF1-α and HIF2-α proteins are the best studied among HIF-α subunits. Each of these subunits contains two proline residues (HIF1-α: P402/P564 and HIF2-α: P405/P531), which are hydroxylated in the presence of oxygen by prolyl hydroxylase domain-containing proteins (PHDs). Hydroxylation of the proline residues promotes binding to von Hippel-Lindau tumour suppressor (pVHL), leading to HIF-α ubiquitination and degradation^4,5^. Another factor regulating HIF-α in an oxygen-dependent manner is factor inhibiting HIF1 (FIH1). Asparagine hydroxylation of HIF1-α (and to a lesser extent, of HIF2-α) driven by FIH1 impedes HIF1 interaction with its cofactors, histone acetylases p300 and CBP, and hence impairs HIF1 transcriptional activity^6–8^. The hypoxic tumour microenvironment (TME) is subjected to HIF-driven transcriptional responses in cancer and stromal cells. In addition, HIF activity switches the cell metabolism into glycolytic mode, increasing glucose consumption and pyruvate, lactate and H+ production. In this review article, we summarise and discuss the influence of hypoxia and HIFs on TME components and how this impacts cancer progression.

## Cancer-associated fibroblasts (CAFs)

It is widely accepted that fibroblasts infiltrating tumour tissue acquire very different features from normal fibroblasts, leading to the definition of CAF. CAFs often represent the major component of tumour stroma, sometimes constituting up to the 80% of the entire tumour^9^. The population of CAFs can be quite heterogeneous, as several progenitor cell types can be reprogrammed into CAFs. Although most CAFs are considered to arise from resident fibroblasts, bone marrow cells, adipocytes, endothelial cells and epithelial cells can also turn into CAFs^10–17^.

Reciprocal paracrine signalling between murine cancer cells and fibroblasts was described by Olaso et al. Melanoma cells could induce proliferation and expression of CAF marker α-SMA in adjacent fibroblasts. These fibroblasts excessively produced glucosaminoglycans and MMP-2, promoting the migration of melanoma cells^18^. Following this initial study, the ability of CAFs to favour tumour progression was shown in a prostate cancer xenograft model when CAFs were co-injected with initiated (tumorigenic) prostatic epithelial cells and promoted their tumorigenic potential, in contrast to co-injection with normal fibroblasts^19^. A study by Bhomwick and Colleagues demonstrated that TGF-beta type II receptor deficiency in mouse fibroblasts led to increased HGF secretion and initiation of tumour formation in adjacent prostate and forestomach epithelium^20^, suggesting one possible mechanism of fibroblast transformation. Other examples of paracrine signalling that is deregulated by CAFs include the secretion of chemokine CXCL12 with subsequent tumour growth facilitation and the expression of intra-cellular and extracellular Ca2+-binding protein S100A4 with subsequent tumour progression and metastatic spread^21,22^. Except for paracrine signalling, the oncogenic functions of CAFs are partially mediated by altered ECM production. In a breast cancer study, ECM deposited by CAFs was organised differently (aligned) than ECM produced by normal fibroblasts and could influence premalignant human mammary epithelial cells, assigning them a mesenchymal phenotype and increasing their tumorigenic and metastatic potential. The mesenchymal phenotype transition in epithelial cells is dependent on the TGF-β-dependent Smad, Erk, Jun and Rho signalling pathways. As TGF-β is stored in the ECM before activation, the function of CAFs in that model could consist of increasing TGF-β availability as well as building an ECM framework with a metastasis-promoting spatial structure^23–26^. In addition to the direct effect of CAFs on cancer cells, they can promote angiogenesis via vascular endothelial growth factor-C (VEGF), CXCL12a and FGF-2 factor production and modulate the immune response by inducing macrophage infiltration and tumour-promoting cell polarisation, reducing T-cell infiltration and interfering with natural killer cell function^27^.

**Fig. 1:**
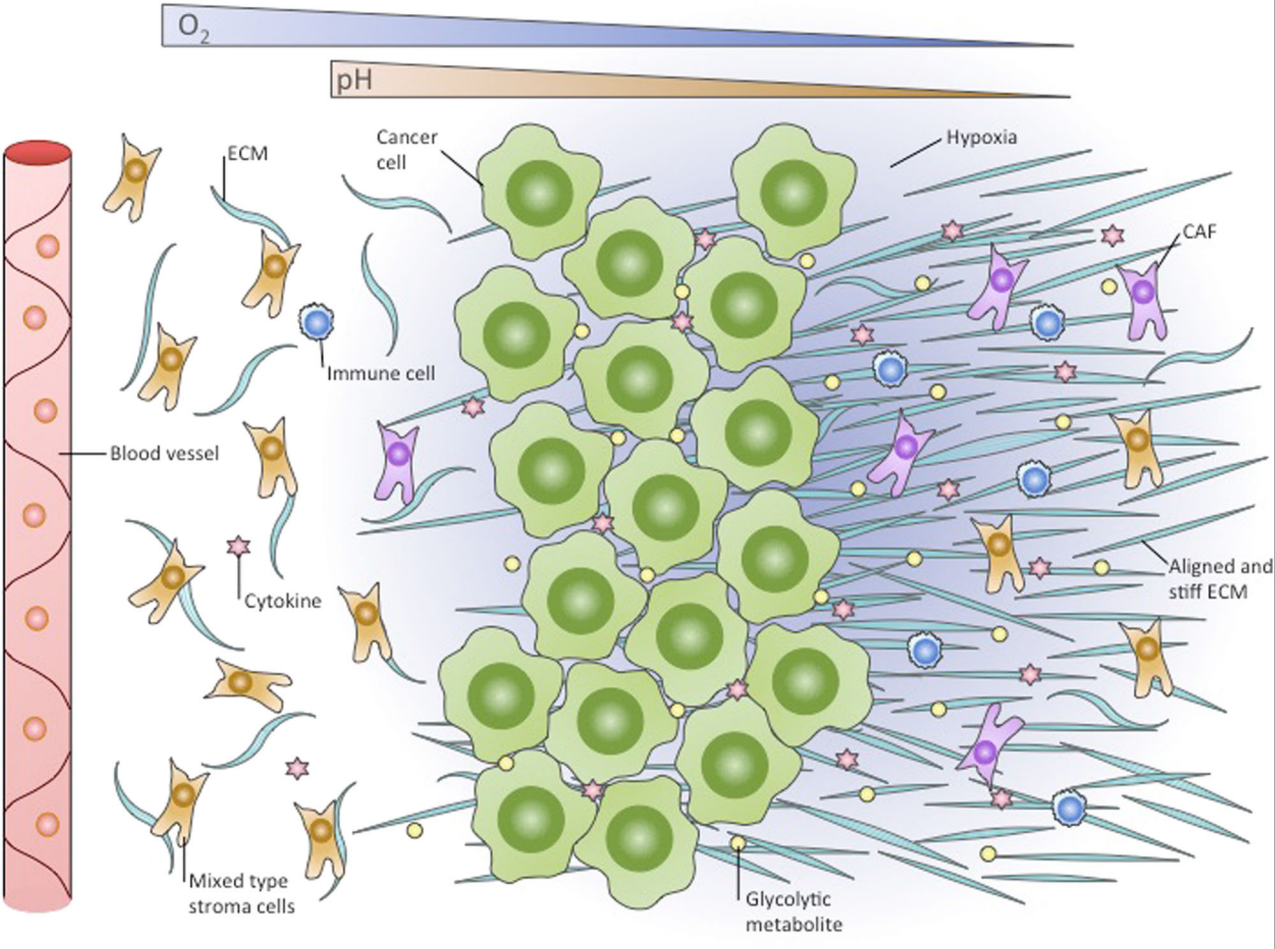
Tumour stroma and extracellular matrix in hypoxia . A rapidly growing tumour leads to a reduction in the oxygen supply of the cancer and in tumour stromal cells that are far from the blood vessels. In hypoxia, these cells switch to glycolytic metabolism, which contributes to the acidification of the tumour microenvironment. Produced glycolytic metabolites such as lactate can be utilised by cancer cells and promote tumour growth. The hypoxic microenvironment is also enriched in diverse types of immune cells, and many of them are recruited from the circulation. Cytokine expression by tumour and stromal cells is altered by hypoxia. In particular, hypoxic cancer cells produce signalling molecules that promote the transformation of fibroblasts into CAFs. Together with cancer cells, in hypoxia, CAFs produce an ECM that is stiff and aligned, different from a normoxic ECM, and support cell migration. CAF cancer-associated fibroblasts, ECM extracellular matrix

Hypoxia can influence both fibroblast reprogramming and tumour-promoting functions (Fig. 1). Oxygen deficiency influences paracrine signalling between cancer cells and fibroblasts. Hypoxia was shown to stimulate cytokine CXCL13 secretion by cancer-associated myofibroblasts in prostate cancer progression^28^. While CAFs secrete chemokine CXCL12, facilitating cancer growth^22^, hypoxia was shown to stimulate CXCR4 (CXCL12 receptor) expression in many cell types, therefore suggesting a feed-forward loop between cancer cells and CAFs^29^. Hypoxic cancer cells can secrete paracrine signalling molecules, which promote reprogramming of progenitor cells into CAFs^30^, and HIF1 was shown to regulate some of these signalling molecules, such as TGF-β, bFGF and PDGF-B^31–33^.

It has been shown that the hypoxia-inducible factor-1 (HIF-1)α level is often upregulated in CAFs. In a model in which CAF formation is stimulated by TGF-β and PDGF treatment, the rate of aerobic glycolysis in primary CAFs was increased compared to that in normal fibroblasts, and this effect was associated with HIF-1α protein stabilisation^34,35^. The lactate produced by highly glycolytic CAFs can be consumed by adjacent cancer cells and lead to induced tumour growth, which suggests a negative outcome of HIF-1 upregulation in fibroblasts^34^. The role of the increase in HIF-1α levels during CAF formation is still poorly understood, that is, whether it is a driving force for reprogramming or a consequence. Chiavarina and colleagues observed that stable HIF-1α overexpression endowed fibroblasts with oncogenic functions, as they increased tumour growth after ectopic co-injection with breast cancer cells. The proposed mechanism included HIF-1α-promoted autophagy, mitophagy, and the production of lactate and recyclable nutrients, which could fuel cancer cells and provide them with building blocks^36–39^. However contradicting evidence supports a negative regulatory role for HIF-1a signalling in stromal fibroblast. Kim and Colleagues indeed showed that selective deletion of HIF-1a (or VEGFA) in fibroblast was enhancing tumour growth in murine mammary cancer models^40^. Additionally after 80 h of hypoxia normal fibroblasts were shown to produce a stiff, aligned matrix, and that this matrix supported the migration of breast cancer cells^41^. Confirming the abovementioned experiments hypoxia was inducing secretion of protumorigenic factors, such as hepatocyte growth factor (HGF), in human fibroblast cell line MRC5 due to HIF-1 activity. Conditioned media from hypoxic MRC5 could promote invasiveness of pancreatic cancer cell line PK8^42^. Thus several studies have shown gain of oncogenic functions by fibroblasts in response to HIF-1 activation, and one can suggest that HIF-1 is able to drive fibroblast reprogramming to CAFs. Seeming controversially to the described studies, Madsen et al. suggest that long-term hypoxia dampens CAF function in a HIF-1-alfa-dependent way. These authors showed that 72 h of hypoxic treatment or 72 h of PHD2 silencing impeded the ability of head and neck CAFs and vulval CAFs to remodel ECM in vivo, which was accompanied by reduced expression of the activated fibroblast marker α-SMA. HIF-1α silencing in these conditions reverted the phenotype. In vivo treatment of breast cancer-bearing mice with PHD-inhibitor DMOG reduced tumour resilience and metastatic potential, while it had no effect on tumour size^43^. This controversy could arise from the fact that CAFs indeed are not identical to normal fibroblasts, originate from different cell types and can develop distinct response. It might also be possible that HIF-1 promotes some of the oncogenic functions of CAFs, such as increasing tumour growth, while inhibiting other oncogenic functions, i.e., metastasis promotion. HIF-2 is known to accumulate and mediate long-term hypoxia responses when HIF-1α is downregulated. Therefore, metastasis inhibition in long-term hypoxia and some other hypoxic effects of CAFs that are currently thought to be HIF-1 dependent indeed may be regulated by HIF-2. HIF-2 functions in CAFs have been poorly assessed and need further exploration. It is worth mentioning that after 80 h of hypoxia, normal fibroblasts produce a stiff, aligned matrix and that this matrix supported the migration of breast cancer cells^41^. Taken together, these studies also raise the possibility of different hypoxic responses in CAFs compared to normal fibroblasts.

## Extracellular matrix

The ECM primarily consists of fibrillar proteins and proteoglycans, which together form a net that serves as a framework for most tissues^44^. Collagens are the dominant component of the ECM and account for approximately 90% of its mass^45^. The physical properties of tumour ECM differ from healthy tissue and continuously change^46–49^. In many cases, solid tumours are characterised by excessive deposition of ECM proteins (fibrosis)^50–55^, and especially by collagen deposition^56–61^. They are the main source for synthesis of ECM proteins, namely collagen, fibronectin and hyaluronan, and on the other hand CAFs are an important source for ECM-remodelling enzymes^62^. CAFs share several features with normal activated fibroblasts, including the ability to produce ECM components, which, on contrary of physiologic microenvironment, results in an abnormal ECM that supports tumour dissemination^63^. ECM and fibroblasts in TME are tightly reciprocally regulated. Modifications of ECM structure or composition induce cytoskeleton reorganisation and signalling cascades in CAFs, further regulating synthesis of ECM components and extracellular remodelling enzymes. Most of these changes in ECM are often supportive for formation of pro-tumorigenic microenvironment^64^. Besides CAFs, cancer cells themselves significantly contribute to ECM remodelling^65^. In breast cancer, localisation of fibrotic areas often coincides with localisation of hypoxic regions^66,67^. HIF1 was shown to directly influence ECM remodelling and promote fibrosis in kidney, liver and adipose tissue^68–70^. After hypoxic treatment, rats have increased mRNA levels of procollagens I, II, and IV^71^. Skin, heart and kidney fibroblasts contain elevated mRNA levels of procollagen I α1 chain when cultivated under hypoxic conditions^72–74^. In fibroblasts, fibulin-5 was shown to be transcriptionally induced by hypoxia or in a PHD inhibitor-induced hypoxic environment in a TGF-β/PI3K/Akt-dependent way, although the role of HIFs in that process was not assessed^75^. Thus, hypoxia may be able to potentiate ECM protein deposition in tumours in a HIF-dependent and HIF-independent manner (Fig. 1).

**Fig. 2:**
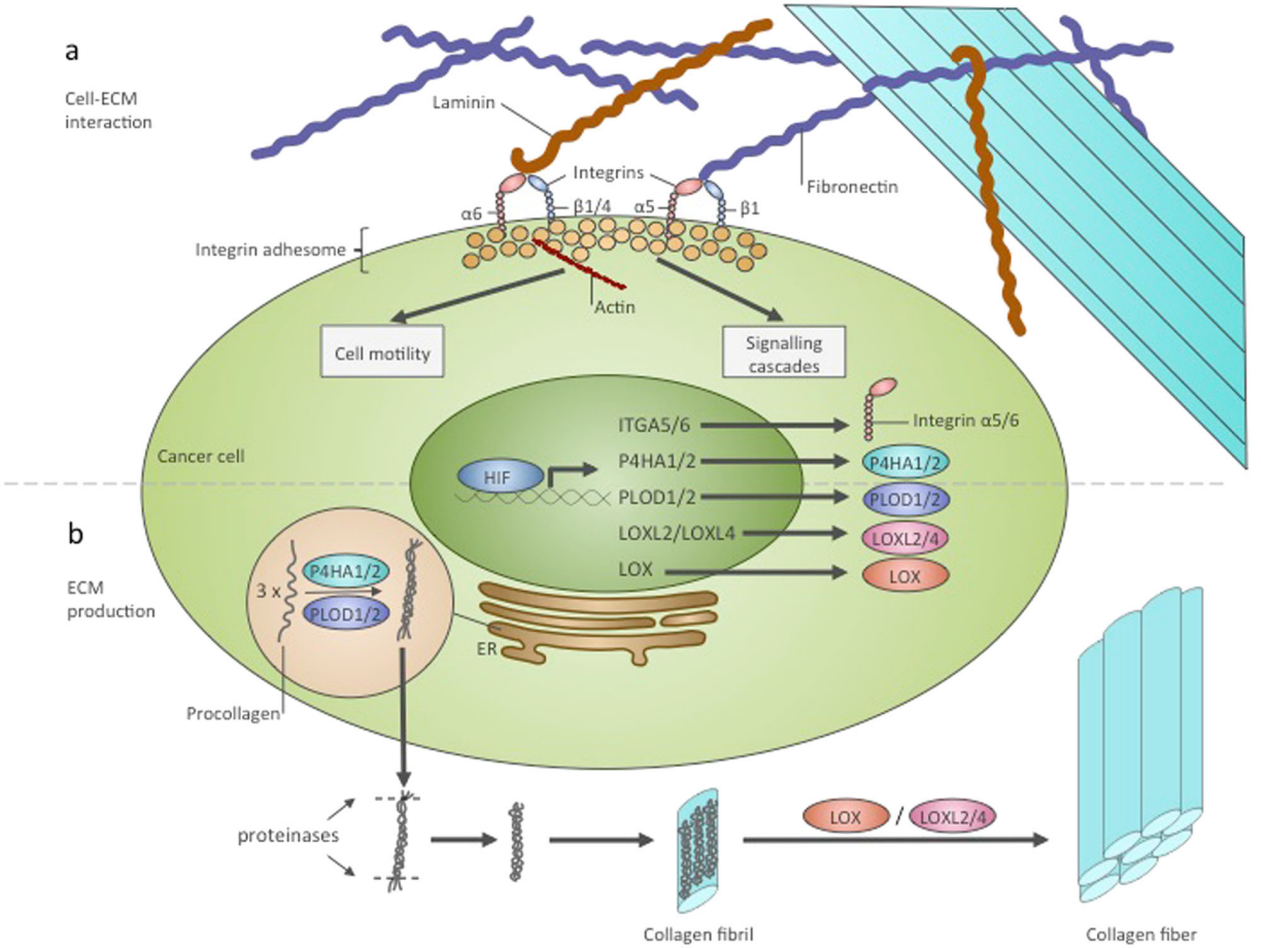
HIF regulates interactions of cancer cells with ECM and ECM biosynthesis . **a** Regulation of cell–ECM interactions by HIF. HIF was shown to transcriptionally induce ITGA5 and ITGA6 genes encoding integrins α5 and α6. Each integrin α subunit together with a β subunit forms a specific ECM receptor. Integrin α5β1 binds fibronectin and integrin α6β1, or α6β4 binds integrin. In the cell, integrins bind with a multi-component complex named the integrin adhesome. Some proteins of this complex can be involved in signalling cascades, and others interact with the cytoskeleton. As a result of interactions with the ECM, cells undergo alteration of their signalling networks and their motility. **b** HIF contributes to collagen production. P4HA1, P4HA2, PLOD1, PLOD2, LOX, LOXL2 and LOXL4 are transcriptional targets of HIF that are involved in collagen posttranslational modification. P4HA1/2 and PLOD1/2 catalyse the first step of procollagen molecule modification, which occurs in the ER and allows the formation of the triple-stranded procollagen molecule. Triple-stranded procollagens are exported from the cell and into the extracellular space, where they are modified by proteinases and assembled in collagen fibrils. Subsequently, LOX, LOXL2 and LOXL4 catalyse the crosslinking of collagen fibrils and the formation of a functional collagen fibre. ER endoplasmic reticulum

The metastatic potential of cancer cells depends on their interactions with the ECM. Cells receive mechanistic signals from the ECM by means of focal adhesions. Formation of focal adhesions requires the binding of ECM proteins with integrins and cell-surface ECM receptors and subsequent signal transduction through intermediate molecules to the cytoskeleton. Silencing of the *ITGA5* gene encoding integrin α5, a subunit of fibronectin receptor α5β1, reduced breast cancer cell motility, migration and invasion capacity. These cells had decreased metastatic potential after orthotopic injection in mice^76^. The same study showed that both HIF-1 and HIF-2 could induce the transcription of integrin subunits α5 and β1. Integrin α6 was demonstrated to be a direct transcriptional target of HIF-1 and HIF-2 and to increase breast cancer cell invasion potential^77^. In addition to integrins, another fibronectin receptor, syndecan-4, is transcriptionally induced in hypoxia by an unknown mechanism^78^. Thus, hypoxia is implicated in the regulation of cell–ECM interactions, partially through HIF (Fig. 2). In addition to integrin expression, hypoxia regulates ECM proteins synthesis. HIFs can regulate collagen production at several stages (Fig. 2). Posttranslational modification of procollagen chains requires prolyl-4-hydroxylases and procollagen lysyl-hydroxylases. HIF-1 can induce the expression of prolyl-4-hydroxylase alfa-subunits P4HA1 and P4HA2 and procollagen lysyl-hydroxylases PLOD1 and PLOD2 in different cancer and non-cancer cell lines^41,79–85^. Expression of P4HA1, P4HA2, PLOD1 and PLOD2 is necessary for the production of stiff and aligned collagen fibrils. In this microenvironment, cancer cells were shown to take on an elongated, adhesive, motile phenotype and have an elevated capacity for invasion and migration^41,81,83,84,86^.

Another point in the collagen deposition process that is regulated by HIFs is the deamination of secreted collagen fibrils on lysine and hydroxylysine residues by lysyl-oxidases. This deamination is required for fibril crosslinking and collagen fibre formation^87^. HIF-1 was shown to induce the expression of lysyl-oxidases LOX^88^, LOXL2^89^ and LOXL4^90^. The LOX family is intimately linked to the metastatic process via several aspects, involving ECM remodelling and potentially ECM-independent EMT regulation^91–93^. The role of LOX expression can be crucial, as lung metastases in a breast cancer model were shown to be dependent on LOX-driven fibrosis^94^. LOX family proteins can mediate several oncogenic HIF-1 functions. LOX knockdown prevented focal adhesion formation and reduced cell motility, abrogating HIF-1-dependent cell migration and invasion^95^. In another study, the transformation of cancer cells to an invasive mesenchymal phenotype was dependent on HIF-1-driven LOX and LOXL2 expression^89^. Hypoxic LOX, LOXL2, and LOXL4 secretion by breast cancer cells results in collagen remodelling in lungs, which allows the recruitment of bone-marrow derived cells (BMDCs) to the area. ECM remodelling performed by LOX and BMDC-derived matrix metalloproteinases favours the formation of pre-metastatic niches, showing that the oncogenic ECM remodelling and tumour cell recruitment driven by hypoxia can also have long-distance effects^88,90,96^.

Simultaneous with collagen synthesis, hypoxia promotes the degradation of extracellular matrix. HIF1 is known to induce the expression of matrix metalloproteinases MMP2^97^, MMP9^98^ and MMP15^99^ and the expression of urokinase receptor uPAR^97^. HIF-2 can increase MMP14 levels^100^. Hypoxia-driven expression of these MMPs can promote invasion and correlates with poor patient prognosis^97,99,101^. Downregulation of tissue inhibitors of metalloproteinases TIMP2 and TIMP3 by hypoxia represents an additional level of hypoxic control over ECM remodelling^98,102^. The simultaneous increases in ECM biosynthesis and degradation, which are driven by HIFs, and the hypoxic regulation of focal adhesion formation creates a mechanism for the metastatic spread of cancer cells.

## Blood vessels

Vascularisation is one of the main outcomes of HIF signalling. HIF-1α and HIF-2α inactivation led to developmental lethality in mice, which was linked to defects in blood vessel formation^103,104^. A connection between excessive vascularisation and cancer was shown in several models. Redundant vessel formation is considered a feature of cancer, and it promotes cancer progression. Some therapeutic approaches aiming to suppress vascularisation have been developed^105–108^. New vessel development was shown to be important for the transition from hyperplasia to neoplasia^109^. Although vessels transport oxygen, they can be affected by hypoxia. The two major components of blood vessels are endothelial cells and pericytes. Those endothelial cells that reside at the end of growing capillaries and direct their branching are located far from functional vessels, and they can become hypoxic and thus develop a hypoxic cellular response^110^. HIF-1α and HIF-2α presence in endothelial cells differentially affects vascularisation. While HIF-1α deletion reduces tumour vascularisation and tumour growth, HIF2-α deletion, on the contrary, is able to augment angiogenesis with the formation of a more disorganised vascular system and more hypoxic tumours^111–113^.

Tumours become hypoxic when they grow too large in size apparently because the blood supply is insufficient. At this point, tumour growth decelerates, and HIF1 promotes the secretion of factors inducing vascularisation from tumour and stromal cells; for example, VEGF influences endothelial cells, pericytes and BMDC to induce vessel growth^114–116^. The ECM produced by cancer cells cultured under hypoxic conditions was also shown to support angiogenic growth^117,118^. HIF-induced neovascularization attempts a compensation of the oxygen deficiency in the tumour tissue. But the rate of uncontrollable proliferation of cancer cells exceeds the speed of organised capillary net formation. Indeed sometimes new blood vessels are able to transiently restore the oxygenation, and in this case cancer tissues have interchanging hypoxic areas with a combination of both acute and chronic hypoxic regions. Fluctuations in red cell flux in tumour microvessels can lead to transient hypoxia and reoxygenation in tumour parenchyma^119^. This uncontrolled activation of hypoxia signalling in tumour mass often results in an aberrant, disorganised vascularisation that fails to compensate oxygen deficiency.

It is noteworthy that endothelial cells play an important role in cancer cell migration, as they are the major structural component of blood vessels and serve as a barrier in extravasation and intravasation processes^120^. HIF1-α depletion in endothelial cells was shown to suppress the migration of tumour cells through endothelial cells, but HIF2-α depletion was shown to stimulate metastatic spread. These opposite effects of HIF1-α and HIF2-α on vessel formation and metastasis can be explained by the ability of these factors to differentially regulate the nitric oxide level, which regulates endothelial cell function^121^.

Pericytes are cells embedded in basement membrane of blood microvessels and cover the endothelial cells. They regulate angiogenesis, but also participate to other functions, such as formation of blood-brain barrier. Hypoxic stress of brain pericytes leads to their migration out of the blood vessels, but at the same time HIF-1-signalling leads to VEGF level upregulation and vascularisation stimulation^122^. During the process of kidney fibrosis detachment of pericytes from the capillaries in response to VEGF and PDGF is followed by their transformation to mesenchymal fibroblasts, actively producing ECM^123^. As both of these factors can be produced in epithelial cells due to HIF-1 signalling, presumably hypoxia in TME could also lead to detachment of pericytes and their subsequent transformation, but this formal experimental evidence in support of this assumption are currently missing.

## Lymphatic vessels

In addition to dissemination in the blood circulation, cancer cells can disseminate through lymphatic vessels. Lymphatic vessel density is increased in breast cancer tissues and correlates with positive lymph node metastases and worsened prognosis^124^. An increased HIF1-α level in primary malignant neoplasias is tightly associated with the density of the surrounding tumour lymphatic vessels and with breast cancer patient mortality^125,126^. In oesophageal cancer, HIF1-α levels correlate with the colonisation of lymph nodes^127^. Different studies suggest a direct contribution of HIF1-α in the regulation of lymphangiogenesis^128,129^. In particular, HIF1-α was proven to induce lymphatic metastases through the activation of different growth factors, including VEGF-c a and PDGF-B^33^. Expression of VEGF-c, one of the major lymphangiogenesis-driving factors, was shown to correlate with HIF1-α expression^130^; however, it remains unclear whether VEGF-c induction requires HIF1-α, as the data are controversial^131,132^. HIF1 contribution to lymphatic vessel formation can be mediated by VEGF-a^133^. In contrast, there is evidence for the opposite role of HIF2-α in lymphangiogenesis, as HIF2-α knockdown increased lymphatic vessel formation in vivo in a xenograft model^134^. The influence of hypoxia on lymphatic vessel formation is a poorly studied topic that needs further investigation, as lymphatic vessel formation contributes to metastatic tumour spread.

## Immune cells

Adaptive immune cells can potentially inhibit tumour growth by the recognition of tumour-specific antigens on the surface of cancer cells and the elimination of those cells. Innate immune cells can promote the antitumour activity of infiltrating lymphocytes and lead to significant tumour regression. HIFs have been shown to be tightly connected with the inflammatory processes^135,136^, and hypoxia can directly or indirectly influence the function of almost all immune cell types, thereby influencing tumour development^137^.

## Innate tumour immunity

Myeloid cell-specific PHD2 depletion was shown to suppress tumour growth and metastases, underlining the importance of oxygen sensitivity for myeloid cells^138^. A hypoxic TME is known to promote neutrophil engagement in tumours by regulation of their adherence to epithelial cells^139,140^. HIF1-α and HIF2-α were separately shown to increase the survival and function of neutrophils^141–144^. The outcome of this hypoxic effect is still unknown, as cancer-associated neutrophils can induce both tumour suppression and tumour progression^145^.

An increase in cancer-associated macrophage density correlates with poor patient prognosis in different types of cancer^146,147^. Macrophage polarisation plays a significant role in tumorigenesis. Indeed, macrophages polarised to the M1 type (classical activation) counteract cancer progression and metastases, while M2-polarised macrophages (alternative activation) can promote it^148^. Hypoxia induces tumour cells to secrete chemoattractants, such as Sema3A, EMAPII, ET-1 and ET-2, promoting the chemotaxis of macrophages from the circulation^149–151^. HIF1-α was shown to be necessary for macrophage maturation, function^141^ and glycolytic reprogramming and when associated with HIF1-α-induced PDK1 activity, it increases the migratory capacity of macrophages, representing a possible mechanism of macrophage infiltration regulation by HIF1-α^152^. In addition, hypoxia determines macrophage polarisation through the induction of M2 polarisation-related genes^153^ and promotes lactic-acid-induced M2 polarisation^154^. In vivo experiments show that HIF1-α and HIF2-α are both crucial for macrophage infiltration and immune suppression in tumours, as their separate ablation led to reduced tumour growth^155,156^.

Another noteworthy innate immune cell is the myeloid-derived suppressor cell (MDSC). These cells originate from bone marrow cells, and their numbers are significantly increased in cancer. Their main feature is the ability to suppress the activity of other immune cells and therefore suppress the antitumour immune response^157^. The role of hypoxia in MDSC regulation has been considered mostly oncogenic. Hypoxia-treated MDSCs show activation of HIF-signalling and of those HIF targets that enhance MDSC function^158^. Hypoxia can also augment MDSC function through a mechanism partially dependent on HIF: by miR-210 regulation and ArgI expression^158,159^. However, some studies suggest that MDSCs are able to gain immunostimulatory properties^160^. Liu and Colleagues described MDSCs’ differentiation into the tumour-suppressing M1 subtype after SIRT1 induction, and this differentiation occurred as a result of mTOR/HIF-1α-dependent glycolytic reprogramming^161^. This observation suggests that HIF1 may also endow MDSCs with tumour-suppressive functions and that the role of HIFs in MDSC regulation needs to be further investigated.

## Adaptive tumour immunity

Although T-cells are able to infiltrate into the tumour, anticancer immunity is often limited due to characteristics of the TME and a hypoxic environment^162^. Increased glycolysis in the tumour, which is partially mediated by HIF activity, is a reason for so-called “metabolic competition” between cancer cells and T-cells. Lack of nutrients suppresses T-cell function and the antitumour response^163–165^. Hypoxia induces the differentiation of non-specific CD4+ T-cells into regulatory T-cells (CD4 + CD25^High^FOXP3+) or T-helpers (Тн17), and expression of transcriptional factors FOXP3 and RORγt, which are crucial for differentiation, is regulated by HIF-1^166–168^. While regulatory T-cells have immunosuppressive functions, the contribution of Тн17 cells to the immune response is unclear^169^. In addition, under hypoxic conditions, cancer cells and macrophages synthesise chemokines and cytokines, which attract regulatory T-cells from the circulation and repress the antitumour response of other T-cells^170–172^. Regulatory T-cells in hypoxia produce extracellular adenosine, which represses effector T-cell function^173,174^. Another mechanism of effector T-cell suppression involves a hypoxia-dependent increase in lysyl oxidase secretion, which causes the formation of premetastatic niches. MDSCs can migrate into those niches and suppress the anti-tumour response of T-killers^95,175,176^.

**Fig. 3:**
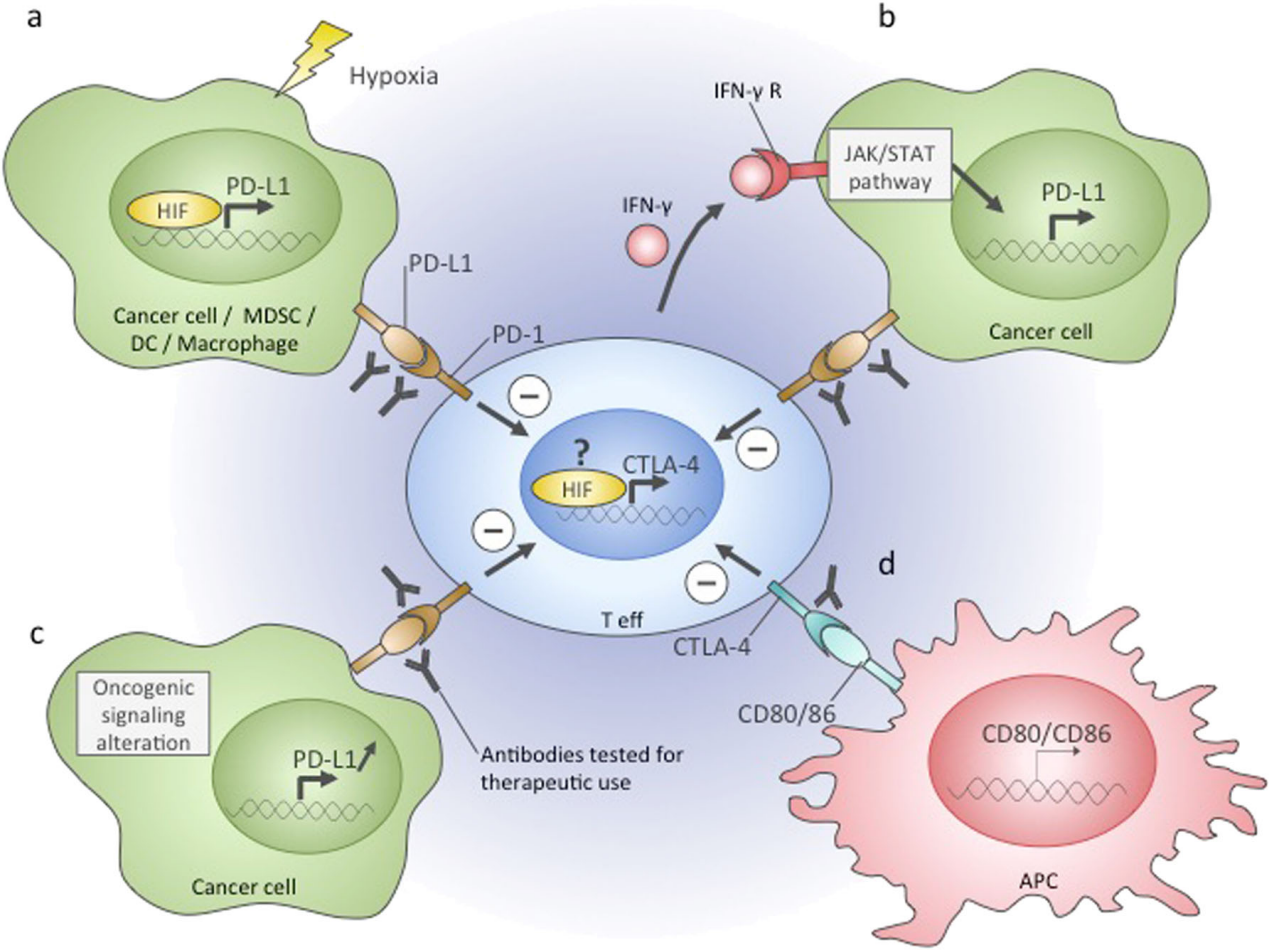
Immune checkpoints in the tumour microenvironment . Effector T cells infiltrating the tumour can become repressed due to the activation of immune checkpoints. The targeting of PD-1 and CTLA-4 checkpoint pathways with specific antibodies is a promising therapeutic approach. In the tumour microenvironment, these pathways can be activated by the following mechanisms. **a** PD-1 receptor binding to its ligand PD-L1 leads to effector T-cell repression. PD-L1 can be expressed on the surface of cancer cells, MDSCs, DCs, and macrophages, and in these cells, it is directly transactivated by HIF in hypoxia. **b** Binding of interferon γ secreted by active effector T cells to its receptor on cancer cells results in activation of PD-L1 gene expression and subsequent T-cell repression. **c** PD-L1 gene expression can be constantly upregulated in cancer cells because of oncogenic mutations and signalling alteration, which leads to effector T cell repression upon interaction with this type of tumour cell. **d** Antigen-presenting cells express CD80 and CD86 ligands. Upon CD80/86 binding to CTLA-4 receptor on T cells, they become functionally repressed. Hypoxia was shown to induce CTLA-4 expression in T-cells, which potentially could contribute to their repression in the hypoxic tumour microenvironment. Teff effector T-cell, MDSC myeloid-derived suppressor cell, DC dendritic cell, IFNγ interferon gamma, IFN-γ R interferon gamma receptor, APC antigen-presenting cell

HIF1 is involved in the regulation of T-cell immune checkpoints (Fig. 3). In cancer cells, macrophages, dendritic cells and MDSCs, HIF1-α directly induces PD-L1 expression^177–179^. Binding of PD-L1 expressed on the cell surface to the PD-1 receptor on T-cells leads to their dysfunction, and hence, this mechanism is a target for different pharmaceutical approaches, such as the development of anti-PD-L1 antibodies and PD-1/PD-L1 interaction inhibitors^180,181^. Another checkpoint regulated by hypoxia is the CTLA-4 receptor, which is upregulated on CD8+ T-cells in hypoxia potentially via HIF1^182^. Binding of CTLA-4 on T-cells to ligands CD80 and CD86 on the surface of antigen-presenting cells results in effector T-cell inhibition and regulatory T-cell activation^183^. As with PD-1, anti-cancer treatments using CTLA-4 blocking antibodies have been developed. CTLA-4 and PD-1/PD-L1-targeted therapies showed positive responses in clinical trials for several types of cancer^184–186^. However, the outcome of the therapies is dependent on many parameters, including the frequency of tumour-infiltrating lymphocytes, which inversely correlates with the glycolytic rate and HIF1-α expression^187,188^.

In contrast to the described effector T-cell suppression by hypoxia, some data indicate that HIF function is important for the activity of effector cytotoxic T-cells. It was shown by Doedens and colleagues in 2013 that elevated HIF-1 and HIF-2 support the function of cytotoxic CD8+ T-cells^182^. In agreement with these findings, a recent study by Tyrakis et al. has suggested a role for HIF1-α in CD8+ T-cell proliferation, differentiation and antitumour activity through the regulation of L-2HG^189^. Apart from cytotoxic T-cells, HIF-1 was also shown to contribute to natural killer cell priming and activation via regulation of the glycolytic rate^190^, thus demonstrating a stimulatory role in immune activation.

## Conclusions

The role of TME in cancer progression is currently attracting impressive interest in the field. Hypoxia is a condition that often occurs at late stages of cancer, and even before that, HIFs can be upregulated due to environmental acidification and the presence of glycolytic metabolites^191–193^. The HIF-mediated hypoxic impact on TME in most cases is mediated by transcriptional activity of HIFs, secretion of signalling molecules by cancer cells and tumour stromal cells, and metabolic changes associated with the switch from oxygen-dependent catabolism to glycolysis. Non-tumorous cells in the TME are all affected by hypoxia and HIFs (Table 1). Often, hypoxia leads to their dysregulation in a way that supports cancer growth: fibroblasts can be transformed into tumour-prone CAFs, ECM remodelling supports metastases, vascularisation process facilitates cancer progression, and antitumour immune function becomes generally repressed. Nevertheless, the hypoxic response can also be detrimental for tumorigenesis (Table 1). This finding can be partially explained by the differences in HIF-1 and HIF-2 stability and function, as acute hypoxic response is mainly mediated by HIF1 activity, and chronic hypoxia by HIF2^194^. Dual roles can also arise from the presence of HIF interactors, among which the p53 family and MDM2 can play important roles, as they can affect HIF stability and function^195,196^. In this context, the interplay between HIF and the p53 family can influence a wide range of cellular processes specifically associated with complexity of p53 family members in controlling cell death^197–201^, metabolism^202–207^, reproduction^208,209^, and development^210–213^.

**Table 1:** Influence of hypoxia on TME components

TME component	Influence of hypoxic environment	Cancer suppression (+)/promotion (−)
CAF progenitors	Recruitment, activation and transformation into CAFs	−
CAFs	Secretion of cytokines, promoting tumour growth	−
	Deactivation by chronic hypoxia	+
ECM	Increase of ECM deposition (fibrosis) and remodelling	−
	Increase of interaction with cancer cells	−
Blood vessels	Increase in vascularisation by HIF-1	−
	Decrease in vascularisation by HIF-2	+
Lymphatic vessels	Increase in lymphangiogenesis by HIF-1	−
	Decrease in lymphangiogenesis by HIF-2	?
Neutrophils	Recruitment to tumour	?
	Increase in survival and function	?
Macrophage	Maturation and functioning are dependent on HIF1	?
	Recruitment from bloodflow	−
	Increase in migratory capacity	−
	M2 polarisation	−
MDSCs	Enhancement of immune-suppressive function	−
	Polarisation to M1 type, driven by SIRT1	+
Non-specific CD4+ cells	Differentiation into regulatory T-cells and T-helpers	−/?
Regulatory T-cells	Recruitment from bloodflow	−
	Enhancement of immune suppressive function	−
Effector T-cells	Immune checkpoints activation	−
	Repression due to “metabolic competition” for nutrients	−
CD8+ T-cells	Support in proliferation, differentiation and activation	+

Considering the global impact of HIF on cancer cells and the TME, therapies targeting HIF activity, such as the usage of small molecules preventing the interactions of the HIF-α and HIF1-β subunits^214^, can be beneficial for some groups of patients.
